# Clinicopathological and biobehavioral factors influence the onset and severity of oral mucositis in patients with head and neck cancer

**DOI:** 10.1007/s00520-026-10679-x

**Published:** 2026-04-29

**Authors:** Monica Moreno de Carvalho, Vitória Parmejane de Oliveira, Giulia Rodrigues Santos, Glauco Issamu Miyahara, Daniel Galera Bernabé, Aline Satie Takamiya

**Affiliations:** 1 Oral Oncology Center, São Paulo State University (UNESP), Araçatuba, São Paulo, Brazil; 2 Department of Diagnosis and Surgery, Araçatuba Dental School, São Paulo State University (UNESP), Araçatuba, São Paulo, Brazil; 3 Oral Oncology Center and Discipline of Integrated Clinic, Araçatuba Dental School, São Paulo State University (UNESP), Araçatuba, São Paulo, Brazil

**Keywords:** Head and neck cancer, Mucositis, Pain, Clinical characteristics, Pathological characteristics

## Abstract

Oral mucositis is a debilitating adverse effect of radiotherapy in head and neck cancer. It progresses from erythema to painful ulcerations, often causing infections, nutritional impairment, treatment interruption, and reduced survival. While treatment-related factors are well established, patient-related risk determinants remain insufficiently defined. Thus, this study investigated the association between the onset and peak severity of radiation-induced oral mucositis in patients with head and neck cancer and their clinicopathological, biobehavioral, and demographic characteristics. To carry out this study, patients diagnosed with head and neck cancer and undergoing mandatory radiotherapy treatment were recruited. Fifty patients undergoing radiotherapy were evaluated daily. The analysis focused on the onset and peak severity of oral mucositis. Chemotherapy was significantly associated (*P* = 0.034) with earlier oral mucositis onset. Diabetes (*P* = 0.017) and radiation dermatitis (*P* = 0.017) were statistically associated with the severity of mucositis at the time of onset, while hypertension (*P* = 0.030), diabetes (*P* = 0.016), mild and moderate alcohol consumption (*P* = 0.029), radiation dermatitis (*P* = 0.001), odynophagia (*P* = 0.022), burning mouth (*P* = 0.003), difficulty with hygiene (*P* = 0.020), and use of opioids (*P* = 0.045) were associated with the most severe degrees at the peak of lesion. Severe smokers were associated with a greater occurrence of mucositis lesions in the lip region at two times. This study demonstrated that the interaction between clinicopathological and biobehavioral factors exhibits distinct patterns depending on the stage of oral mucositis progression. A comprehensive understanding of these complex relationships is crucial for developing targeted strategies to prevent severe mucositis and optimize treatment outcomes.

## Introduction

Oral mucositis (OM) is a common adverse effect of radiation therapy in patients undergoing treatment for head and neck cancer (HNC) [1]. Radiation-induced OM typically develops more slowly and requires a longer healing period [2]. Lesions often emerge within two weeks after the initiation of radiotherapy (RT), initially presenting as erythema of the oral mucosa accompanied by a burning sensation. As the condition progresses, these lesions can evolve into deep, intensely painful, confluent ulcerations that markedly elevate the risk of opportunistic infections [3, 4]. The cellular changes associated with radiation-induced OM manifest clinically as debilitating pain, dysphagia, dysgeusia, decreases in weight, and factors that increase susceptibility to secondary systemic infections [5–7]. Furthermore, the severity of OM may necessitate the placement of feeding tubes, hospitalization, and the suspension of oncologic treatment [8]. Treatment interruption can significantly impair therapeutic outcomes, increase the possibility of cancer recurrence, and decrease overall survival, ultimately compromising patients’ quality of life across physical, psychological, and social well-being [4, 9].

Despite its high incidence and significant clinical impact, the risk factors for OM in patients with HNC remain poorly defined. The incidence and severity of mucosal lesions vary according to the intensity and fractionation schedule of RT, as well as the regimen of chemotherapeutic agents [10, 11]. Notably, hyperfractionated RT and the concomitant administration of conditioning regimens and chemotherapy (CT) have been associated with increased risk, severity, and duration of mucosal damage [12]. Patients undergoing CT concomitant with RT have a risk of approximately 90–100% of developing OM at some stage of antineoplastic treatment [7]. However, patient-related risk factors remain unclear. Variables such as age, sex, body mass index, alcohol and tobacco consumption, pre-existing dental conditions, disease stage, salivary flow, and alterations in oral microbiota have been suggested as potential contributors to OM, but the available data supporting their relevance are limited and inconclusive [13–15].

Patients exhibited varying responses to treatment, underscoring the need to investigate contributing clinical, biobehavioral, and demographic factors. Therefore, the primary objective of this study was to assess the association between the onset and peak severity of radiation-induced OM in patients with HNC and their clinicopathological, biobehavioral, and demographic characteristics.

## Patients and methods

### Study design and ethical aspects

The Institutional Research Ethics Committee approved this study under protocol 72346123.3.0000.5420. Patients who agreed to participate in the research signed a Free and Informed Consent Term. Participants were informed about the purpose of the study and were assured of confidentiality and anonymity. All participants volunteered without any subsequent reward.

### Characterization of the sample and the study

Ninety-eight patients were selected for an initial consultation, which included an analysis of their medical records, information about the study and written informed consent. Fifty adult patients (aged > 18 years), all of whom had started RT treatment with curative intent, were included in the sample. The convenience sample consisted of individuals treated at Oral Oncology Center (COB), an auxiliary unit of São Paulo State University (UNESP), School of Dentistry, Araçatuba, as part of an observational, prospective and longitudinal clinical study. The assessments were conducted by assessors who had been previously calibrated for the clinical evaluation of the criteria analyzed in this study. The patients included in this study underwent a clinical protocol, which consisted of preparation of the oral environment with dental cleaning, check-up and prior extraction of compromised teeth. In addition, patients were instructed on the possible transient and permanent side effects in the mouth of antineoplastic treatment with RT.

The inclusion criteria were patients diagnosed with HNC, located in the oral cavity, oropharynx, or salivary glands, patients aged > 18 years, undergoing radiation treatment and followed up daily in our oncology patient support center. The exclusion criteria consisted of individuals with cognitive impairment and inability to participate in the study, who had contraindications to RT, who had a history of RT, who did not follow the daily follow-up at our center, who did not finish the RT treatment, or who refused to sign the informed consent. All patients underwent 3D conformal RT, which consists of shaping the radiation beam to the region to be treated based on three-dimensional images from computed tomography, magnetic resonance imaging and a daily protocol of 2 Gy of radiation, not exceeding a total dose of 70 Gy.

### Daily evaluation

The laser prophylaxis was carried out on all participants, daily performed with the Therapy EC (DMC®, São Carlos, SP, BRA). The following parameters were used daily from the first session until the end of the RT sessions in the regions without OM: total diode laser energy 1 J (per point) for 10 s with 1 cm distance between points, wavelength of 660 nm, and application in regions free of tumor lesions. After the first clinical sign of OM, the following photobiomodulation (PBM) protocol was included in the regions with OM lesions: total diode laser energy 2 J (per point) for 20 s, wavelength 660 nm, prioritizing the repair of superficial tissue and pain control. All participants were followed up on the PBM therapy until complete resolution of the signs and symptoms of OM.

OM was classified by the World Health Organization (WHO) [16], assigning scores: (0) no mucositis; (1) irritation or erythema; (2) erythema and ulcerative lesions that still allow a solid diet; (3) ulcerative lesions in which the patient is restricted to a liquid diet; (4) when oral feeding is not possible. According to the grading scale, grades 1–2 were considered the mild group and grades 3–4 were the severe group. Oral pain was analyzed subjectively using a Visual Analogue Scale (VAS), where “0” is the absence of pain and “10” is the maximum pain experienced by the patient. Patients were instructed to assign a score to the degree of oral pain. For the primary statistical analyses, a binary categorization was employed: absence of pain (0) and presence of pain (1). Concurrently, for descriptive purposes and secondary analyses, an ordinal scale was utilized, classified as: (0) no pain; (1) mild pain (1 to 3); (2) moderate pain (4 to 7); and (3) severe pain (8 to 10).

Data on feeding route (oral or nasogastric tube), type of feeding (solid, paste, or liquid), medications, or presence of oral alterations such as odynophagia, dysphagia, dysgeusia, xerostomia, burning mouth, and difficulty with oral hygiene were collected daily from the patients. A previously trained dental surgeon assessed the presence of oral candidiasis or radiation dermatitis. Radiation dermatitis was classified according to the Radiation Therapy Oncology Group (RTOG) [17] scale based on the degree of toxicity, which indicates the severity of the condition. Medications were classified as analgesics (no opioids) or opioids, systemic or topical antifungals. Other medications such as antibiotics, anti-inflammatories, or antacids were only classified as use or nonuse. 

### Demographic and clinical-pathological characteristics

Demographic and biobehavioral characteristics such as sex (female or male), age (adult patients 45–65y or elderly > 65y), marital status (single, married, divorced or widowed), ethnicity (white, brown or black), smoking and alcohol consumption, were collected directly from the patients and from their medical records before the cancer diagnosis was notified. Tobacco and alcohol consumption were assessed using a scale of mild to severe score according to the number of cigarettes and alcoholic drinks consumed per day. Smoking: mild, less than 20 paper cigarettes or up to 4 straw cigarettes; moderate, 20–39 paper cigarettes or 5–7 straw cigarettes; severe, 40 or more paper cigarettes or 8 or more straw cigarettes. Alcoholism: mild, 1 dose of distilled alcohol or 1 bottle of beer; moderate, 2–3 doses of distilled alcohol or 2–3 bottles of beer; severe, 4 or more doses of distilled alcohol or 4 or more bottles of beer.

The clinical-pathological characteristics included the presence of comorbidities, type of oncologic treatment, clinical staging, and tumor stage. The staging of HNC followed the criteria of TNM [18], and the N was also classified as (N-) without lymph node metastases and (N +) with the presence of lymph node metastases. The evidence of distant metastasis (M) was not classified, as none of our patients had distant metastasis. The clinical stages were classified as I, II, III, and IV.

### Data analysis

Statistical analysis was performed using Statistical Package for the Social Sciences (SPSS) software (version 24.0, NY, USA). All data collected were tested for normality before analysis. Descriptive data analysis was performed using mean and standard deviation (SD). The data were tested by the chi-square test or Fisher’s exact test; these tests were used to determine any statistically significant association between different parameters among different variables. Two distinct phases of OM were subjected to rigorous analysis: the onset and the peak severity. These periods were strategically chosen to determine the demographic, biobehavioral, and clinicopathological factors associated with the initiation of OM and the subsequent progression to its maximal severity. The first phase begins approximately 1 to 2 weeks after the start of RT [1], and the peak is between weeks 2 and 3 of treatment [19]. Consequently, the analysis periods were defined as sessions occurring before and after the 10th fraction for OM onset, and sessions before and after the 15th fraction for peak severity. The value of *P* ≤ 0.05 was considered statistically significant.

## Results

A total of fifty adult patients participated in the study. Seventy-eight percent of the patients were male, with a mean age of 64.74 years (range, 45–84 years). Regarding ethnicity, 70% of participants were white. In terms of marital status, 58% were married, 20% divorced, 14% single, and 8% widowed. Concerning general health, 88% of the patients had at least one comorbidity before cancer diagnosis, with hypertension reported by 46%, gastritis by 26%, and diabetes mellitus by 22%. The majority of patients (94%) were diagnosed with oral squamous cell carcinoma (OSCC). The most common tumor site was the oral cavity (58%), followed by the oropharynx (36%) and the salivary glands (6%). Tumor staging revealed that 8% of patients were classified as T1, 24% as T2, 48% as T3, and 20% as T4. Additionally, 40% presented lymph node involvement (N +). Clinical staging showed that 8% were in stage I, 16% in stage II, 48% in stage III, and 20% in stage IV. As for treatment, 76% of the patients underwent RT combined with CT, with or without surgical intervention (Table 1).

**Table 1:** Demographic and clinicopathological variables of patients

Variable	Patients
	No. (%)
Age	
Mean age (years, SD)	64,74 (SD 9,76)
Adults 45-65y	24 (48.0)
Elderly > 65y	26 (52.0)
Sex	
Male	39 (78.0)
Female	11 (22.0)
Ethnicity	
White	35 (70.0)
Brown	11 (22.0)
Black	4 (8.0)
Marital status	
Single	7 (14.0)
Married	29 (58.0)
Divorced	10 (20.0)
Widowed	4 (8.0)
Comorbidity	
Yes	44 (88.0)
No	6 (12.0)
Comorbidity type	
Hypertension	23 (46.0)
Diabetes	11 (22.0)
Gastritis	13 (26.0)
Types of cancer	
OSCC	47 (94.0)
Others	3 (6.0)
Cancer localization	
Oral cavity	29 (58.0)
Oropharynx	18 (36.0)
Salivary gland	3 (6.0)
T classification	
T1	4 (8.0)
T2	12 (24.0)
T3	24 (48.0)
T4	10 (20.0)
Regional metastasis	
N0	30 (60.0)
N +	20 (40.0)
Clinical stage	
I	4 (8.0)
II	8 (16.0)
III	24 (48.0)
IV	14 (28.0)
Treatment	
Concomitant chemotherapy	38 (76.0)
Without concomitant chemotherapy	12 (24.0)

Abbreviations: *OSCC*, oral squamous cell carcinoma

Regarding lifestyle habits, 46% of the patients were smokers, 36% were ex-smokers, and 18% had never smoked, and regarding alcohol, 42% of the patients were current drinkers, 32% were former drinkers, and 26% had non-drinkers. In terms of consumption intensity, 22% were mild smokers, 38% moderate, and 20% severe, while about alcohol, 20% were mild drinkers, 28% moderate, and 18% severe alcohol consumption (Table 2).

**Table 2:** Biobehavioral variables of patients

Variable	Patients
	No. (%)
Smoking	
No	9 (18.0)
Current smoker	23 (46.0)
Ex-smoker	18 (36.0)
Tobacco intensity*	
Non-smoker	9 (18.0)
Mild	11 (22.0)
Moderate	19 (38.0)
Severe	10 (20.0)
Alcohol consumption	
Non	13 (26.0)
Current drinker	21 (42.0)
Ex-drinker	16 (32.0)
Alcohol intensity*	
Non-drinker	13 (26.0)
Mild	10 (20.0)
Moderate	14 (28.0)
Severe	9 (18.0)

^*^Variables with missing data

Tobacco intensity: mild: less than 20 paper cigarettes or up to 4 straw cigarettes; moderate: 20–39 paper cigarettes or 5–7 straw cigarettes; severe: 40 or more paper cigarettes or 8 or more straw cigarettes. Missing data for tobacco intensity: *n* = 1. Alcohol intensity: mild: 1 dose of distilled alcohol or 1 bottle of beer; moderate: 2–3 doses of distilled alcohol or 2–3 bottles of beer; severe: 4 or more doses of distilled alcohol or 4 or more bottles of beer. Missing data for alcohol intensity: *n* = 4

Upon analyzing the clinicopathological associations at the onset of OM, a significant association was identified between patients who had started to show signs of OM before the tenth RT session and those who received concomitant CT (*P* = 0.034). There was a significant association between patients who presented OM lesions after the tenth RT session and odynophagia (*P* = 0.043). The severity of the OM was significantly associated with diabetes (*P* = 0.017), radiodermatitis (*P* = 0.017), and mild degrees of radiodermatitis (*P* = 0.013). Heavy smokers had more OM lesions in the lip area (*P* = 0.001) than other smokers or nonsmokers (Table 3).

**Table 3:** Associations of clinicopathological and biobehavioral characteristics in the onset of OM

Variables	Qualitative assessment (*P-Value*)
	OM before the 10th session
Chemotherapy	**0.034**
Trismus	0.418
	OM after the 10th session
Radiation dermatitis	0.074
Odynophagia	**0.043**
Dysgeusia	0.074
Analgesics	0.719
Topical and systemic antifungal	0.384
	Severity of the OM
Hypertension	0.585
Diabetes	**0.017**
Mild or moderate alcohol consumption^*^	0.444
Radiation dermatitis	**0.017**
Odynophagia	0.373
Mouth burning	0.501
	Lip mucositis
Severe smokers^*^	**0.001**

^*^Variables with missing data

Missing data for alcohol intensity: *n* = 4. Missing data for tobacco intensity: *n* = 1

The peak severity of OM up to the fifteenth RT session was not significantly associated with any variable. The variables significantly associated with peak severity of OM after the fifteenth session were the presence of radiodermatitis (*P* = 0.000), odynophagia (*P* = 0.020), dysgeusia (*P* = 0.023), use of analgesics (*P* = 0.044), and topical and systemic antifungals (*P* = 0.005). The severity of OM was associated significantly with hypertension (*P* = 0.030), diabetes (*P* = 0.016), mild and moderate alcohol consumers (*P* = 0.029), radiodermatitis (*P* = 0.001), odynophagia (*P* = 0.022), burning mouth (*P* = 0.003), oral hygiene difficulties (*P* = 0.020), and the use of opioids (*P* = 0.045). In addition, patients classified as severe smokers also had more OM lesions in the lip area (*P* = 0.017) (Table 4).

**Table 4:** Associations of clinicopathological and biobehavioral characteristics in peak severity of OM

Variables	Qualitative assessment (*P-value*)
	Peak severity of OM before the 15th session
Chemotherapy	0.565
Trismus	0.064
	Peak severity of OM after the 15th session
Radiation dermatitis	**0.000**
Odynophagia	**0.020**
Dysgeusia	**0.023**
Analgesics	**0.044**
Topical and systemic antifungal	**0.005**
	Severity of the OM
Hypertension	**0.030**
Diabetes	**0.016**
Mild or moderate alcohol consumption^*^	**0.029**
Radiation dermatitis	**0.001**
Odynophagia	**0.022**
Mouth burning	**0.003**
Hygiene difficulties	**0.020**
Opioids	**0.045**
	Lip mucositis
Severe smokers^*^	**0.017**

^*^Variables with missing data

Missing data for alcohol intensity: *n* = 4. Missing data for tobacco intensity: *n* = 1

There was no significant association between OM and the variables of age, gender, color, marital status, use of medication, gastritis, tumour size (T), lymph node involvement (N +), staging, total dose (Gy), presence of pain, candidiasis, route of feeding, type of feeding, dysphagia, xerostomia, use of anti-inflammatory and use of antacids in both periods evaluated (*P* ≥ 0.05).

## Discussion

The results of this clinical study, which evaluated the association between clinicopathological, biobehavioral, and demographic factors with the onset and period of peak severity of radiation-induced OM in patients with HNC, showed a significant association between several factors and the two stages of OM. To our knowledge, no other studies have related these factors to the onset and peak of radiation-induced OM. The prediction of OM risk has been a subject of ongoing scientific investigation. Broadly, the risk factors can be categorized into three main groups: (1) treatment-related factors, (2) patient-related factors, and (3) tumor-related factors [13]. Regarding treatment-related factors, in the present study, it was observed that a higher incidence of OM lesions emerged by the tenth RT session among patients who received concurrent CT, compared to those who underwent exclusive RT. Consistent with these findings, several studies have demonstrated that the concomitant administration of CT and RT significantly contributes to the earlier onset and increased severity of OM lesions [8, 20, 21]. While the addition of CT to standard radiation protocols has been associated with improved tumor response, it has also been linked to a nearly threefold increase in the incidence and severity of OM-related toxicity [22]. The severity of OM lesions has also been related to the accumulated radiation dose, the dose fractionation regimen, the type of radiation, and the volume of irradiated tissue [23]. Higher daily doses of radiation (daily fractions of up to 3.5 Gy) in accelerated fractionation regimens have been shown to lead to the development of more severe OM lesions, which is the main reason for treatment interruptions and prolonged total treatment time [23, 24]. In our study, all patients received daily fractions of 2 Gy, not exceeding a total of 70 Gy. There were no significant association results between the severity of OM and patients who underwent less than 60 Gy or more than 60 Gy of total radiation.

The Clinical Practice Guidelines established by the Multinational Association of Supportive Care in Cancer and International Society of Oral Oncology (MASCC/ISOO) stipulate that basic oral care must encompass all routine patient- or caregiver-performed actions aimed at reducing the oral bacterial load and, consequently, preventing infections. Furthermore, the implementation of multi-agent combination oral care protocols is considered beneficial for the prevention of OM during RT. For the prophylaxis of OM in patients with HNC undergoing treatment with RT or RT-CT, therapeutic agents such as anti-inflammatory mouthwashes (e.g., benzydamine), natural compounds (e.g., honey), and oral glutamine have been suggested. Regarding the management of OM-associated pain, topical 0.2% morphine mouthwash is recommended for HNC patients receiving RT-CT to enhance patient comfort [25]. Moreover, the use of intraoral PBM therapy, specifically low-level laser therapy, is advocated for both the prevention and treatment of OM in adults undergoing RT for HNC [25, 26]. At our institution, established oral hygiene guidelines and protocols are strictly adhered to both prior to and throughout oncological treatment. This regimen is supplemented by dedicated PBM monitoring. For symptomatic relief, patients presenting with pain are counseled to utilize topical and systemic analgesics.

OM is frequently associated with pain in the oral and/or oropharyngeal mucosa, increased opioid consumption, significant discomfort, and functional impairments in eating and swallowing, collectively exerting a substantial negative impact on patients’ quality of life [24, 27, 28]. In the present study, interestingly, no association was found between oral pain and OM; however, there was a significant association between the severity of OM and the use of opioids, odynophagia, and burning in the mouth at the peak severity of radiation-induced lesions. The factors that could explain these results are the fact that the opioid drugs are masking the patients’ pain and the use of PBM, which can help to achieve an analgesic effect on the oral lesions [29]. PBM, particularly utilizing low-level laser therapy (LLLT), exerts well-documented anti-inflammatory, analgesic, and tissue-healing properties. Mechanistically, PBM involves the interaction of specific light wavelengths with biological tissues, leading to the modulation of various metabolic processes. This process transforms light energy into cellular energy, effectively modulating the inflammatory cascade within physiological limits, thereby mitigating active symptoms associated with OM, including pain [30]. It is imperative to acknowledge that all patients in the present study received daily prophylactic PBM as part of the institutional standard of care. Although PBM is a well-established intervention for mitigating the incidence and severity of OM, its ubiquitous application within this cohort constitutes an inherent confounding factor. Consequently, the observed outcomes are representative of a population receiving systematic prophylactic laser therapy, which may constrain the generalizability of these findings to clinical settings where PBM is not routinely implemented for HNC patients undergoing RT.

A study by Morais-Faria et al. [31] identified a higher incidence and severity of OM and trismus in younger patients during sessions 21 and 35 of RT. Our findings did not indicate an association between trismus and the peak of OM lesions, specifically after the fifteenth RT session, with no statistically significant relationship between trismus and patient age. Moreover, the severity of OM lesions after the fifteenth session of RT was significantly associated with the presence of odynophagia, increased use of analgesics, and the administration of topical and systemic antifungal agents. These findings may reflect elevated levels of pain and a heightened risk of opportunistic infections after cumulative radiation doses exceeding 30 Gy. In addition, the peak severity of OM was significantly associated with the presence of radiation dermatitis at both times. Radiation dermatitis is one of the most common side effects of RT and can cause pain and discomfort [32].

Factors associated with the patient, such as poor oral hygiene, alcohol consumption, smoking, xerostomia, opportunistic infections, sex, microbiome, and certain comorbidities, can also influence the severity and incidence of radio-induced OM [13, 14, 33]. Our results showed a significant association between peak severity of OM and difficulty with oral hygiene. Concerning the oral microbiome, it is well established that bacterial colonization of ulcerated lesions in radiation-induced OM can exacerbate the inflammatory response, thereby delaying wound healing and lesion resolution [20, 34–36]. The presence of food debris contributes to the creation of an acidic oral environment, which may further facilitate microbial imbalance and lesion persistence [37]. Several studies have shown that dysbiotic changes in the oral microbiota can influence the onset, progression, and severity of OM, and that interindividual variability in microbial composition may be associated with differences in clinical presentation and disease trajectory [3, 38, 39].

Regarding smoking, our data showed that patients classified as heavy smokers exhibited more frequent and localized OM lesions on the lips during both the onset and peak severity of OM. The relationship between smoking and OM during RT remains inconclusive. While some studies reported no significant association between tobacco use and acute radiation-related toxicities, including OM, others suggested a protective role [40, 41], whereas additional research indicated an increased risk of complications associated with smoking [3, 11, 42]. Alcohol consumption is another factor known to compromise mucosal integrity by increasing epithelial permeability and impairing tissue repair mechanisms [43]. In our study, patients classified as mild and moderate alcohol consumers had more severe degrees of OM during the peak period of lesions. These outcomes suggest that alcohol may negatively affect wound healing processes, thereby enhancing the toxicity of HNC treatments.

Patients with diabetes mellitus had more severe OM at the onset of lesions and at the peak in our study. Diabetes mellitus can cause changes in the microvasculature of the oral mucosa, making it more susceptible to radiation damage and hindering the healing process [44]. Additionally, common oral conditions in diabetic individuals, such as periodontal disease and xerostomia, may further predispose this population to the development of OM and exacerbate its associated symptoms [45]. Diabetes can also compromise immune function, thereby facilitating the emergence of oral lesions and increasing the risk of secondary infections. These lesions may serve as portals of entry for pathogens, contributing to increased severity and delayed recovery [46]. Regarding hypertension, the current literature provides limited evidence on its relationship with radiation-induced OM. For instance, Tao et al. [11] found no significant association between hypertension and acute OM in patients undergoing RT for HNC. However, in our study, a significant association was observed between hypertension and the severity of OM during the peak of lesions, suggesting a potential role for this comorbidity in influencing OM progression, which justifies further investigation.

Sex has increasingly been investigated as a potential factor influencing the risk of developing radiation-induced OM. Several studies have reported that female patients may be at a higher risk than males [3, 47, 48], while another study suggests susceptibility among male patients [49]. However, Sonis 2022 [13] emphasized that data on sex as a risk factor for OM in patients with HNC remain limited, and the current evidence is inconclusive. In the present study, all participants developed OM lesions, and no statistically significant differences were observed between lesion severity and sex.

The main limitations of this study include the relatively small sample size and the unequal distribution of participants by sex, with a predominance of male patients. The statistical analysis also relied on a univariable approach, which may not fully account for potential confounding factors. Additionally, details about the specific chemotherapeutic agents administered were not available, preventing analysis of the possible influence of various CT regimens on the severity and progression of OM.

The present investigation, while providing important data regarding the association between clinicopathological, biobehavioral, and demographic variables and the onset and peak severity of radiation-induced OM in patients with HNC, is subject to several methodological constraints that must be acknowledged. A primary limitation stems from the relatively small sample size and the absence of a sample size calculation, which, coupled with the unequal distribution of participants by sex (with a notable predominance of male patients), may limit the generalizability of the findings. Furthermore, although multivariate analysis was attempted to account for potential confounding factors and complex interactions, the statistical analysis ultimately relied on a univariable approach. This decision was necessitated by challenges in multivariate modeling, including unstable 95% confidence intervals, primarily due to the relatively small sample size. Thus, to ensure statistically reliable and clinically interpretable results, the univariable approach was retained. Furthermore, the universal administration of prophylactic PBM across the entire sample serves as a potential confounding variable, limiting the external validity of the results to populations where such preventive measures are not standard practice. A critical drawback is the absence of a comprehensive microbiological assessment, a necessary element given the discussion concerning intestinal dysbiosis and its potential influence on outcomes. Additionally, the mandated daily clinical monitoring introduces the risk of an observer effect bias, potentially skewing observed data. Another significant constraint is the lack of detailed information regarding the specific chemotherapeutic agents administered, which precluded an analysis of the possible impact of various CT regimens on the severity and progression of OM. Finally, the evaluation of treatment impact is restricted by the absence of patient-reported outcome measures, such as standardized quality-of-life (QoL) scores, thereby limiting a holistic assessment from the patient’s perspective.

## Conclusions

The interaction among clinicopathological and biobehavioral factors demonstrated distinct patterns depending on the stage of OM. Therefore, a comprehensive understanding of the association among these variables and OM is an essential development of targeted interventions aimed at minimizing severe treatment-related toxicity. Such knowledge is critical to improving the quality of life and overall clinical outcomes for patients with HNC undergoing RT.

## Data Availability

Data is provided within the manuscript or supplementary information files.
